# Bioactive Compounds from Pigmented Corn (*Zea mays* L.) and Their Effect on Health

**DOI:** 10.3390/biom14030338

**Published:** 2024-03-12

**Authors:** Yaír Adonaí Sánchez-Nuño, Martín Zermeño-Ruiz, Olga Deli Vázquez-Paulino, Karla Nuño, Angélica Villarruel-López

**Affiliations:** 1 Department of Wellbeing and Sustainable Development, Northern University Center, University of Guadalajara, Colotlán 46200, Mexico; yair.sanchez@academicos.udg.mx; 2 Department of Pharmacobiology, University Center for Exact and Engineering Sciences, University of Guadalajara, Guadalajara 44430, Mexico; martin.zermeno@academicos.udg.mx (M.Z.-R.); olga.vazquez@academicos.udg.mx (O.D.V.-P.); 3 Department of Psychology, Education and Health, Nutrition, ITESO Jesuit University of Guadalajara, Guadalajara 45604, Mexico

**Keywords:** pigmented corn, *Zea mays* L., functional foods, nutraceuticals, bioactive compounds, phytochemicals, chronic noncommunicable diseases, antioxidants

## Abstract

Pigmented corn is a gramineae food of great biological, cultural and nutritional importance for many Latin American countries, with more than 250 breeds on the American continent. It confers a large number of health benefits due to its diverse and abundant bioactive compounds. In this narrative review we decided to organize the information on the nutrients, bioactive compounds and phytochemicals present in pigmented corn, as well as their effects on human health. Phenolic compounds and anthocyanins are some of the most studied and representative compounds in these grasses, with a wide range of health properties, mainly the reduction of pro-oxidant molecules. Carotenoids are a group of molecules belonging to the terpenic compounds, present in a large number of pigmented corn breeds, mainly the yellow ones, whose biological activity incorporates a wide spectrum. Bioactive peptides can be found in abundance in corn, having very diverse biological effects that include analgesic, opioid and antihypertensive activities. Other compounds with biological activity found in pigmented corn are resistant starches, some fatty acids, phytosterols, policosanols, phospholipids, ferulic acid and phlobaphenes, as well as a great variety of vitamins, elements and fibers. This review aims to disseminate and integrate the existing knowledge on compounds with biological activity in pigmented corn in order to promote their research, interest and use by scientists, nutrition professionals, physicians, industries and the general population.

## 1. Introduction

Corn (*Zea mays* L.) is part of the biological and cultural heritage of several Latin American countries, including Mexico. It is currently classified into more than 250 breeds on the American continent, while 59 are known in Mexico. A large number of varieties are derived from these breeds, the result of selection and improvement processes carried out by the peasant communities that adopted them thousands of years ago, mainly in the Mesoamerican region [1]. Phytochemicals of corn have received less attention than these of fruits, vegetables and other grains. The consumption of corn and other whole-grain products has been linked to the reduced risk of chronic diseases including cardiovascular disease, type 2 diabetes, obesity, some cancers and with the improvement of digestive tract health [2].

Currently, corn is grown in different shapes and kernel shades, such as blue, black, cherry, purple and red, known as pigmented corn. Pigmented corns are those that, unlike white corn, contain phenolic and/or carotenoid compounds (terpenic compounds) in one or more of the kernel structures: pericarp, aleurone and/or endosperm. These terpenic and phenolic compounds (mainly anthocyanins) give the kernels shades ranging from blue–violet to almost black, to red. Pigmented maize is appreciated in the producing communities and is used to prepare traditional dishes; however, it is at risk of disappearing due to the decrease in the cultivation area, its limited use and its low demand as a raw material for corn mills. In view of this, strategies are being sought to conserve them, promote their planting and increase their production in the field [3].

For this review, the search for scientific articles was carried out in the search engines and electronic bookstores PubMed, Google Scholar, ScienceDirect, Dialnet, SciELO, Latindex, Redalyc, Medigraphic and Elicit using the keywords in English and Spanish: pigmented corn, blue corn, red corn, black corn, pigmented corn and health, pigmented corn and human health, pigmented corn and health benefits, blue corn composition, red corn composition, yellow corn composition, black corn composition, purple corn composition, *Zea mays* benefits and *Zea mays* and human health. Additionally, various key words were used to relate the pathologies with the highest prevalence and incidence of the different functional compounds found in pigmented corn. The selection of articles was carried out from December 2022 to January 2024 with the following criteria: (a) articles in English and Spanish published from 1980 to 2023 (this wide time range is due to the low number of scientific articles that clinically study the effects of pigmented corn and their bioactive compounds on human health), (b) original articles, narrative reviews, systematic reviews and systematic reviews with meta-analyses written in English and Spanish, (c) theses and dissertations published in repositories of public and private universities in Spanish, (d) official websites of national and international organizations and public and private universities in Spanish and English and (e) studies directly related to the search objective.

Within the limitations of the review study, the following results were obtained: a reduced number of clinical trials were carried out with bioactive compounds obtained from pigmented corn, most meta-analyses with the reviewed molecules were used on preclinical studies, limited data from meta-analyses with these pigmented corn molecules in clinical studies, wide variability in the reports of the clinical outcomes of the bioactive compounds of interest found in pigmented corn, a lack of information on the adverse effects of several of the bioactive compounds analyzed and a lack of composition studies of pigmented corn. Due to this, we have also decided to use information on bioactive compounds found in corn but extracted from other plant species in order to increase the information shown in this review. This review aims to analyze the clinical evidence of the bioactive compounds found in pigmented corns to encourage readers, researchers and clinicians to study the understudied bioactive compounds derived from pigmented corn.

Table 1 summarizes the bromatological composition of pigmented corn reported by various authors [4,5,6,7]. In general, the composition among the different pigmented corns, as well as in comparison to white and yellow corns, may present different ranges of variation. Bromatologically, pigmented corn tends to contain a higher percentage of carbohydrates, while proteins and lipids are higher in white corn. Likewise, all macronutrients are lower in yellow corn. Several studies have been conducted on the composition of pigmented corn. In blue corn, the results were as follows: protein 7.99%, crude fat 4.24 g/100 g and fatty acids (g/100 g): palmitic 14.66, stearic 3.53, oleic 41.54, linoleic 38.34 and linolenic 1.15. For red corn: protein 8.20%, crude fat 4.05 g/100 g of corn oil and fatty acids (g/100 g): palmitic 13.48, stearic 3.23, oleic 38.19, linoleic 42.82 and linolenic 0.95 [8]. It is important to mention that the content of the phytochemicals, micronutrients and macronutrients varies according to the variety of corn, its race (genotype), as well as the season in which it was grown and harvested. This gives each breed and variety a different nutraceutical potential and largely determines its possible uses in the food, pharmaceutical and cosmetic industries, among others.

Pigmented corns contain anthocyanins in different ranges, depending on the breed and variety, which give them colorful colors, as well as carotenoid compounds (carotenes and xanthophylls). These phytochemical compounds give pigmented corn a nutraceutical advantage over white and even yellow corn, despite the latter’s high carotenoid content [3]. Anthocyanins can be found in the pericarp and aleurone, or only in one of them. Pigments have also been observed in its endosperm [9]. The location of the pigments is of great importance because it is an indication of the amount of pigments that can be extracted and allows one to decide on the best way to use these compounds without degrading them [3]. Other groups of compounds found in pigmented corn, although in smaller quantities, are free phenols, non-anthocyanidic flavonoids, ferulic acid, lutein, zeaxanthin, cryptoxanthin, alpha and beta carotenes, among others.

## 2. Phenolic Compounds and Anthocyanins in Pigmented Corns

Phenolic compounds or polyphenols are compounds resulting from the secondary metabolism of plants, with more than 8000 known molecules [10]. Although several classifications have been made, the most widely used divides polyphenols into two main families: flavonoids (e.g., chalcones, flavones, flavonols, flavandiols, anthocyanins, condensed tannins and aurones) and non-flavonoids (e.g., free phenols, phenolic acids, polyphenolic ketones, fumarins, chromones, benzofurans, lignans, xanthones, stilbenes and quinones) [11]. Phenolic acids are a class of secondary metabolites highly distributed in plants. According to their chemical structure, phenolic acids can be divided into benzoic and cinnamic acids. The main benzoic groups are gallic, pro-tocatechinic and *p*-hydroxybenzoic acids, mainly as conjugates. Cinnamic acids are widely distributed in plants, as esters or amides. The most representative are caffeic, chlorogenic and ferulic acids [12]. Table 2 shows the contents of phenolic compounds quantified in corn of different colors, which vary widely from one another. However, pigmented corns, as well as their by-products, tend to contain significantly higher amounts of these compounds [13]. Phenolic compounds include anthocyanins and anthocyanidins of various types, ferulic acid and phlobaphenes.

Cereals, including corn, are the most important source of ferulic acid, derived from cinnamic acid (intake ranges from 0.092 to 0.32 g) [14]. Ferulic acid ([E]-3-[4-hydroxy-3-methoxyphenyl] propa-2-enoic acid) belongs to the phenolic acid group, commonly found in plant tissues [15]. Phenolic acids are secondary metabolites of variable chemical structures and biological properties. The antioxidant mechanism of action of ferulic acid is complex, based mainly on the inhibition of the formation of reactive oxygen species (ROS) or nitrogen, but also on the neutralization of free radicals. In addition, this acid is responsible for chelating protonated metal ions, such as Cu(II) or Fe(II) [16]. Ferulic acid is not only a free radical scavenger, but also an inhibitor of enzymes that catalyze the generation of free radicals and an enhancer of the activity of scavenger enzymes. It is directly related to its chemical structure. It has also been shown to have lipid peroxidation inhibitory activity [17]. Ferulic acid has low toxicity and possesses many physiological functions, including anti-inflammatory, antimicrobial, anticancer (e.g., lung, breast, colon and skin cancer), antiarrhythmic and antithrombotic activity. It also demonstrated anti-diabetic effects and immunostimulant properties, as well as reduced nerve cell damage, and may help repair damaged cells. [18].

Ferulic acid has been shown to have an angiogenesis effect by affecting the activity of the main factors involved, i.e., vascular endothelial growth factor (VEGF), platelet-derived growth factor (PDGF) and hypoxia-inducible factor 1 (HIF-1) [18]. In research with human umbilical vein endothelial cells, ferulic acid has been shown to enhance VEGF and PDGF expression and increase the amount of hypoxia-induced HIF-1, which generates responses to hypoxia [19]. Ferulic acid appears to be an effective substance that promotes the formation of new vessels, as demonstrated in in vivo and in vitro studies [20].

It is important to note that in corn, ferulic acid can be found bound to arabinoxylans, a class of carbohydrates consisting of arabinoses and xyloses, both five-carbon monosaccharides (pentoses) [16]. Several studies have shown that dietary supplementation with cereal-derived arabinoxylans improves the antioxidant capacity of intestinal epithelial cells due to the production of ferulic acid and short-chain fatty acids (SCFA) from microbial fermentation. Ferulic acid may co-operate with SCFA to regulate intestinal integrity and host immune functions. Peroxisome proliferator-activated receptor γ (PPARγ) may play an important role in the integration of ferulic acid and SCFA to regulate host health and metabolism [21]. In other studies, ferulic acid has been shown to combine with arabinose residues in cereal-derived arabinoxylans, but gut microbiota ferment arabinoxylan to release free ferulic acid, as well as SCFA production [22]. It has also shown that as one of the phenolic acids it has a strong antioxidant capacity to scavenge reactive oxygen species (ROS) and enhance anti-oxidant activity through the activation of the Kelch-like ECH-associated protein 1 and nuclear factor E2-related factor 2 (Keap1-Nrf2) signaling pathway [23]. Therefore, the pericarp of pigmented corn, rich in ferulic acid, could be metabolized by the intestinal microbiota of humans, generating a release of ferulic acid bound and conjugated into free ferulic acid, in a manner similar to thermal, acidic and alkaline processes.

Another phenolic compound found in some pigmented corn, specifically in the red breeds and varieties, is phlobafen. These are condensed tannins of a high molecular weight, coming from the union of several molecules of naringenin and eriodictyol joined by the central ring. They are oxidized, hardly soluble in water—probably due to the abundance of methoxyl groups in their structure—and present a reddish-brown color. There are also phlobaphenes composed of a mixture of polymeric procyanidins, dihydroquercetin, carbohydrate (glucosyl) and methoxyl moieties [24]. In the case of red corn, these accumulate in the pericarp of the seed and the glumes of the cob. A study showed that they are related to the thickness of the pericarp of red corn (the higher the amount of phlobafen, the thicker the pericarp) [25]. It is speculated that they could have beneficial effects on human health due to their high antioxidant capacity; however, up to this moment there are no clinical trials that confirm this effect. The biological effects of phlobafen are still unknown, so there is a great opportunity for future research to elucidate the effects of these phytochemicals and their biological activity in human physiology.

In order to identify pigmented corn with nutraceutical potential, a study analyzed the content of secondary metabolites, phenolic compounds, the antioxidant capacity and the antimutagenic activity of red and blue corn. The ranges of the total phenol, flavonoid and anthocyanin contents of the corn samples were 69.4 to 212.8 mg gallic acid eq./100 g DW (dry weight), 0.07 to 12.19 mg (+) catechin eq./100 g DW and 3.89 to 34.17 mg cyanidin-3-O-glucoside eq./100 g DW, respectively [26,27]. The phenolic extracts demonstrated the highest antioxidant capacity, evaluated by the ABTS assay, showing values from 2.06 to 7.34 mmol Trolox (vitamin E equivalents)/100 g DW. The total phenol and anthocyanin contents correlated with the observed antioxidant capacity. The corn samples with the highest biological activity were those with a blue color, while the least active were those with a red color. The results showed that the blue corn samples studied are good sources of antioxidant and antimutagenic compounds that could be used to develop products that contribute to human health [26,27].

One of the most important flavonoids are anthocyanins. These are water-soluble pigments, abundant in nature, which can be found in vegetables, fruits, flowers and grains. Chemically, they are glycosides of anthocyanidins, i.e., they consist of an anthocyanidin molecule to which a sugar is attached by a β-glucosidic bond. Anthocyanins can be formed from two metabolic biosynthetic pathways: the shikimate pathway to produce the amino acid phenylalanine and the malonyl-CoA pathway (polyacetates or acetyl-CoA pathway) [28]. In purple corn kernels, as in wheat and barley, anthocyanins are found in the pericarp, while in blue varieties they are found in the aleurone layer [29]. In black and dark red grains, anthocyanins are found in both the aleurone and pericarp layers [30,31]. Table 2 summarizes the content of phenolic compounds and anthocyanins in various presentations of corn of different colors [8,13,32].

**Table 2:** Ranges of phenolic compounds and anthocyanins content in various presentations of corn of different colors [8,13,32].

Parameters	Blue Corn	Corn High in Carotenoids	Red Corn	White Corn	Yellow Corn
Free phenols (mg GAE/100 g)	39.1–45.5	40.3–53	26.4–38.2	34.7–47.2	41.5–51.1
Bound phenols (mg GAE/100 g)	95.5–220.7	108.6–270.1	85.3–205.6	97.3–226	102.1–242.2
Free ferulic acid (µg/100 g)	683–17,587	970–21,566	588–8202	455–9988	645–17,462
Conjugated ferulic acid (µg/100 g)	1451–31,746	1965–37,743	1259–29,391	756–15,588	1474–27,827
Bound ferulic acid (µg/100 g)	23,330–127,851	40,610–150,077	22,865–128,450	20,470–119,201	44,527–100,849
Total anthocyanins (mg CGE/100 g)	2.63–6.87	0.56–4.63	2.08–9.75	0.28–1.33	0.29–0.57

CGE: cyanidin-3-O-glucoside equivalents; GAE: gallic acid equivalents. Table adapted mainly from the work of [13]. Corn presentations can include whole kernels, dough, flour, tortillas and fried. Thermal processes such as cooking can significantly increase the amount of some free phenolic compounds, as well as decrease the flavonoid content.

The daily intake of flavonoids and anthocyanins has been reported to be around 200–250 mg/day [33], while the Food and Drug Administration and NHANES (National Health and Nutrition Examination Survey of the United States) have set it at 12.5 mg/day/person [34,35,36,37,38].

Several in vitro assays, animal and human cell line studies, animal models and human clinical trials indicated that the consumption of anthocyanin-rich foods (among which are pigmented corn), beverages and supplements provides numerous health benefits. In fact, this is due to the easy ability of anthocyanins to scavenge and/or neutralize free radicals and reactive species, chelate metals, control signaling pathways, decrease pro-inflammatory markers and thus reduce the risk of cardiovascular pathologies, cancer and neurodegeneration [39].

Anthocyanins have demonstrated antioxidant potential in both in vitro [40] and in vivo studies [41] and the consumption of anthocyanin-rich foods has been linked to lower risks of chronic diseases [42,43,44]. There are several mechanisms of action through which anthocyanins could exert their biological effects on human health, among which is the activation of nuclear factor erythroid 2 (Nrf2). It serves as a transcription factor for the expression, transcription and translation of the antioxidant response element (ARE), which encodes for several antioxidant enzymes, including superoxide dismutase (SOD), glutathione peroxidase, catalase, etc., [45]. Another way in which anthocyanins exert their antioxidant power is by donating hydrogenions, thus reducing a large number of pro-oxidant molecules, as well as neutralizing various free radicals. This is due to the hydroxyl groups in anthocyanins, which usually contain between two and three of these. The last mechanism described by which they can exert an antioxidant and thus anti-inflammatory effect is through the chelation of metals and metalloids, mainly transition metals such as iron (Fe), copper (Cu), nickel (Ni), aluminum (Al), cadmium (Cd) and arsenic (As), as well as their respective valence forms [31]. The anthocyanins identified in several varieties of peruvian pigmented corns are cyanidin-3-glucoside, pelargonidin-3-glucoside, peonidin-3-glucoside, cyanidin-3-(6″ malonylglucoside) and cyanidin-3-(3″,6″-dimalonylglucoside) [30].

Delphinidin, a type of anthocyanidin that can act as a precursor of many anthocyanins, shows the most considerable ability to scavenge superoxide species, followed by petunidin > malvidin = cyanidin > peonidin > pelargonidin, at 1 µM. Similar results were obtained for the ability of these compounds, at the same concentration, to scavenge peroxynitrite radicals [46]. In addition, cyanidin 3-*O*-glucoside at concentrations between 100 and 200 µM showed potential to protect human keratinocyte HaCaT cells against ultraviolet-A radiation, preventing DNA fragmentation and hydrogen peroxide (H_2_O_2_) release [39,47]. In one study, 12 healthy participants who consumed one of two beverage options high in anthocyanins and anthocyanidins, composed of 165.9 mg/L and 303.8 mg/kg of anthocyanins, respectively, showed increases in their plasma antioxidant capacity by 3-fold and 2.3-fold, respectively [48].

Anthocyanins decrease plasma low-density lipoproteins (LDL), leading to a reduction in their accumulation in the walls of medium and large arteries [49]. Thus, anthocyanins indirectly inhibit LDL-promoted endothelial cell activation/dysfunction. Endothelial damage affects the nitric oxide (NO) release which, together with the increased local degradation of NO by the increased generation of reactive oxygen species (ROS), decreases NO availability. Anthocyanins can increase NO availability by several mechanisms. After activation, the endothelium begins to express cell adhesion molecules on its surface (ICAM-1, intercellular adhesion molecule-1 and VCAM-1, vascular cell adhesion molecule-1) to recruit circulating monocytes to the site of oxidized LDL (oxLDL) accumulation. Anthocyanins downregulate the expression of these adhesion molecules [50]. On the luminal side, anthocyanins decrease chemokines (CK), which also results in decreased myeloid cell recruitment. Anthocyanins counteract ROS on both the luminal and intimal sides, reducing LDL oxidation in the vessel wall [51]. During the progression of atherogenesis, neutrophil-derived granular proteins stimulate macrophage activation to a proinflammatory state that can be inhibited by anthocyanins [52]. Both the antioxidant and anti-inflammatory effects of anthocyanins decrease foam cell formation. Anthocyanins also lower cholesterol by reducing its accumulation in the lipid-rich necrotic core [41]. During the late stages of atherosclerosis, anthocyanins reduce the expression of Toll-like receptor 2 (TLR2) signaling in endothelial cells that regulate the neutrophil stimulation of stress and endothelial cell apoptosis [53]. Regarding anthocyanin-enriched fractions of natural products, extracts of blackberries, blueberries, strawberries, sweet cherries and red raspberries at 10 µM showed the potential to inhibit human LDL oxidation, having been twice as effective as an ascorbic acid control [39,54]. Blackberry and raspberry fruits also revealed lipid peroxidation inhibitory potential, showing IC_50_ values below 50 µg/mL [55].

Anthocyanins are also involved in the regulation of the inflammatory status and activation of endogenous antioxidant defenses, as well as in the regulation of the immune system through MAPK-, NF-κB- and JAK-STAT-related signaling pathways [50]. The effects of anthocyanins on inflammatory markers are promising and may have the potential to exert anti-inflammatory biological action in vitro and in vivo. Therefore, translating these research findings into clinical practice would effectively contribute to the prolonged maintenance of a healthy state. A review study summarized the results of clinical studies from the last five years in the context of the anti-inflammatory and antioxidant role of anthocyanins in a health state as preventive agents and concluded that there is evidence indicating that anthocyanin supplementation in the regulation of proinflammatory markers among the healthy population is highly functional, although inconsistencies between the outcome of randomized controlled trials (RCTs) and meta-analyses were also noted. Regarding the effects of anthocyanins on inflammatory markers, there is a need for long-term clinical trials with large cohorts that allow the quantifiable progression of inflammation [56].

In another study, different anthocyanin dilutions (concentrations of 100, 150 and 200 µg/mL) showed the ability to reduce the expression levels of cyclooxygenase-2 (COX-2), inducible nitric oxide synthase (iNOS), IL 1β and IL -6 and to suppress AP-1 signaling and nuclear factor kappa B (NF-kB) pathways [57]. It was also verified that, at concentrations of 10, 25 and 50 µg/mL, they can decrease the phosphorylation of IKK, IkBa, p65 and JNK, prevent the nuclear translocation of p65 in RAW 264.7 macrophage cells stimulated with LPS/IFN-γ and inhibit lipoxygenase activity [58]. These biological activities demonstrate the direct and indirect antihypertensive, anti-inflammatory endothelial vasodilator enhancement and modulation of inflammasome activation, as well as other signal transduction pathways related to the immune response.

In another study, the phenolic profile and associated antioxidant properties of corn samples with different pigmentations were characterized using spectrophotometric and chromatographic techniques and the stability of anthocyanins during gastrointestinal digestion was evaluated. Pigmented varieties showed a significantly higher anthocyanin content compared to common yellow varieties and, as a consequence, higher antioxidant activity. However, although corn is among the cereals mostly used in gluten-free products, it can produce an inflammatory response in some people with gluten intolerance. Therefore, after chemical characterization, the safety of pigmented varieties for patients with gluten intolerance was confirmed by different in vitro models (a cell agglutination test and measurement of transepithelial electrical resistance). Although in vivo studies are necessary, the data collected in the aforementioned study underline that pigmented corn could play a role in reducing oxidative stress at the intestinal level [59]. Cellular assays applied in another study confirmed the absence of alterations by pigmented strains in the permeability of the cell monolayer, a key step in the mucosal inflammatory cascade in various intestinal disorders [60]. Considering the daily consumption of corn and the high content of anthocyanins in pigmented corn, these varieties could contribute antioxidant and anti-inflammatory ingredients to the diet of the general population, but in particular, of people with gastroenteric disorders since corn represents one of the most important ingredients among the cereals used in the formulation of gluten-free products [61].

In a study, monomeric anthocyanin contents and antioxidant activity were determined by DPPH and TBARS methods, as well as the in vitro antiproliferative activity of blue corn and blue corn tortillas. The tortilla anthocyanin profile was obtained by HPLC-ESI-MS. The antiproliferative activity of the tortilla and blue corn extract on human cell lines Hep-G2 (hepatocellulatar carcinoma), H-460 (lung cancer), HeLa (cervical cancer), MCF-7 (breast cancer) and PC-3 (prostate cancer) was evaluated by an MTT assay [62]. Blue corn had a higher monomeric anthocyanin content, as well as a lower percentage of polymeric color than the tortilla; however, both showed similar antioxidant activity by DPPH. Also, although higher anthocyanin degradation was observed in the tortilla extract, both extracts inhibited lipid peroxidation (IC50) at a similar concentration. The anthocyanin profile showed 28 compounds derived mainly from cyanidin, including acylated anthocyanins and proanthocyanidins. Blue corn and tortilla extracts showed antiproliferative effects against HepG2, H-460, MCF-7 and PC-3 cells at 1000 μg/mL; however, Hela cells had higher sensitivity at this concentration [62]. Anthocyanins activate the MAPK molecular signaling cascade, in turn activating MEKK1/4, which in turn activates MKK3/6, resulting in the activation of the nuclear transcription factor p38 [63].

Another trial described some red and blue pigmented maize in terms of their secondary metabolite content and antioxidant and antimutagenic properties. High concentrations of ferulic acid were found for both red and blue corn, while the cyanidin-3-O-glucoside content was prominent for blue corn. Likewise, blue corn samples proved to be good sources of antioxidant and antimutagenic compounds, mainly those belonging to anthocyanins. These pigmented maize can be considered for scaling up production to obtain natural dyes, bioactive extracts for pharmaceutical and cosmetic applications and maize-based products that contribute to human health [26].

There is some evidence from in vitro, animal and human studies supporting the beneficial effect of cereal-based anthocyanins on a variety of health outcomes such as obesity, diabetes, aging, cancer and cardiovascular disease (Table 3). However, more research is needed to determine the true effects of anthocyanins in humans. In addition, most studies used purified extracts to the test health effects. However, this is an unrealistic means of consuming cereal-based anthocyanins. More trials are needed to elucidate the effect of anthocyanin consumption within a matrix of processed cereals, including those made from pigmented corn [31]. Table 3 shows the most studied biological effects of phenolic compounds and anthocyanins found in pigmented corn in humans, based on clinical trials and systematic reviews with meta-analyses.

## 3. Carotenoids in Pigmented Corn

Carotenoids are organic pigments of the isoprenoid group found naturally in plants and other photosynthetic organisms such as algae, some kinds of fungi and bacteria; they belong to the group of terpene compounds or terpenoids, being tetraterpenes. More than 700 compounds belonging to this group are known to exist [70]. Various carotenoids, both carotenes and xanthophylls, are present in pigmented corn, being found in greater quantities in yellow corn [4].

Lutein and zeaxanthin belong to the xanthophyll family of carotenoids, pigments produced by plants. Key sources of these carotenoids include kale, collard greens, spinach, broccoli, peas, parsley, corn and egg yolks. Yellow corn is rich in carotenoids (up to 823 μg/100 g DW corn) including lutein (50%), zeaxanthin (40%), β-cryptoxanthin (3%), β-carotene (4%) and α-carotene (2%) [2,71]. The recommended daily intake of lutein is approximately 10.0 mg, while that of zeaxanthin is 2 mg. Lutein intake in adults varies, with an average intake of 1–2 mg/day. Due to the lack of synthesis of the intake of these compounds in humans, these substances are extremely important for the proper functioning of certain organs of the body (eye, skin, heart, intestines, etc.).

Maize presents a higher carotenoid content compared to non-corn cereals, with zeaxanthin as the dominant carotenoid [72]. The protective effects of carotenoids are mainly related to their defense against oxidative stress and their ability to scavenge free radicals. Lutein and zeaxanthin are the only dietary carotenoids that accumulate in the retina, specifically in the macula, and are called macular pigments. These carotenoids are concentrated by the action of specific binding proteins such as StARD3, which binds lutein, and GSTP1, which binds zeaxanthin and its dietary metabolite, mesozeaxanthin. It has been shown that supportive therapy with lutein and zeaxanthin may have a beneficial effect in delaying the progression of eye diseases such as age-related macular degeneration (AMD) and cataracts [73].

The possibility of using modern research techniques may provide new evidence for the effective role of lutein and zeaxanthin in the etiology of age-related macular degeneration (AMD). So far, treatment options for the dry form of AMD are limited and all efforts are limited to inhibiting progression to advanced forms of this degeneration, i.e., geographic atrophy and exudative form. The AREDS 2 study, conducted between 2006 and 2012, developed a dietary supplement formulation that can reduce the risk of progression to advanced forms at certain stages of the disease. This risk is reduced by about 25% in people with moderate disease in both eyes or moderate disease in one eye and advanced disease in the other eye. The research continues on other treatments for dry AMD, including methods using nanosecond laser-2RT, orally administered drugs (emixustat) or intravitreal preparations (lampalizumab, sirolismus, pegcetacoplan, etc.) [74,75,76]. So far, the results of the use of these forms of therapy are not satisfactory. Other forms of treatment, independent or complementary, are also being sought. The effects of inhibiting other growth factors, such as angiopoietin (Faricimab) [77], the use of a Port Delivery System (PDS) with ranibizumab [78] and gene therapy [79] are being tested. It appears that in aging retinal tissue, the inhibition of the endogenous antioxidant capacity, marked by a decrease in macular xanthophylls (lutein, zeaxanthin and mesozeaxanthin), is an important factor contributing to the progression of AMD. The use of adjuvant therapy with carotenoid phytochemicals in clinical treatment algorithms for AMD appears to be warranted. It has been shown that adjunctive therapy with carotenoid phytochemicals not only provides neuroprotection, but may also have a beneficial effect on treatment strategies at any stage of AMD, even in advanced AMD [73]. Table 4 shows the most studied biological effects of carotenoids found in pigmented corn in humans, based on clinical trials and systematic reviews with meta-analyses.

## 4. Bioactive Peptides and Inflammation

Bioactive peptides (BPs) are generally a group of peptides, in most cases consisting of less than 50 residues, that have a function in a living organism or cell. Although some of these peptides are in naked form, many of them are hidden in the intact structure of protein molecules [85]. The content of BP chains in most cases comprises the amino acids proline, arginine and lysine, together with hydrophobic residues [86]. In terms of quantity, the protein content of maize kernels is composed mostly of prolamins or zeins (40%), followed by glutelins (30%), with globulins and albumins found in lesser quantities (5%) [87,88]. Zeins are mainly found in protein bodies in the rough endoplasmic reticulum and constitute ~44–79% of maize endosperm proteins. Zeins are devoid of lysine and tryptophan, amino acids that are essential for human survival [88].

From a structural point of view, there is no consensus on the architecture of BPs [89]. They are classified into two main types: endogenous and exogenous peptides. Endogenous peptides are produced in different cell types, such as neural cells (analgesic/opioid application) or immune cells (role in inflammation and antimicrobial), or in various glands throughout the body, such as the pituitary and adrenal glands. Exogenous peptides enter the body from various sources, such as food, dietary supplements and drugs [86,90]. Notably, certain activities have recently been discovered in plant-derived peptides that may perform important functions in humans; among these benefits are anti-diabetic, immunomodulatory, antimicrobial, hypocholesterolemic, opioid, antihypertensive and antioxidant activities [86].

The presence of hydrophilic amino acids such as proline, alanine, valine and leucine in the N-position and the amino acids tyrosine, valine, methionine, leucine, isoleucine, glutamine and tryptophan in the C-terminal position was associated with the antioxidant properties of peptides [91]. In addition, liposoluble free radicals (peroxyl radicals) produced during the oxidation process of unsaturated fatty acids are neutralized by hydrophobic amino acids such as leucine, valine, alanine and proline [92]. Amino acids such as histidine, tyrosine, methionine and cysteine inactivate free radicals by providing them with protons. Aromatic amino acids (phenylalanine, tryptophan and tyrosine) convert free radicals into stable molecules by providing them with electrons [86,93].

Most food-derived antioxidant peptides include hydrophobic amino acids such as valine or leucine at the N-terminal end and proline, histidine, tyrosine, tryptophan, methionine and cysteine in their sequence. Hydrophobic amino acids such as valine or leucine can increase the affinity of peptides in the fatty phase, thus facilitating access to free radicals produced in the fatty phase [86,94].

A wide range of plant-derived peptides can help diabetics through a variety of pathways. The pathways that have been studied so far include inhibitory properties on alpha-amylase, dipeptidyl peptidase IV, the glucose transporter system and mimicking insulin activity [86,95].

Antimicrobial peptides are probably involved in all stages of host defense. Apart from enhancing the immune response, these compounds prevent uncontrolled inflammation by suppressing proinflammatory responses. Despite the specific overlap, antimicrobial peptides interact with each other, complementing each other to guide effective cells to the site of inflammation and modulate the local immune response [96]. Phagocytes, neutrophils and monocytes adsorb through alpha-defensins, human neutrophil peptides 1HNP1-3 and beta-defensins such as human β-defensins 2hBD3 and 3hBD4, whereas mast cells adsorb through HNP1-3, LL-37 and 4B. Likewise, hBD1 and hBD3 are chemotactic for immature dendritic cells and memory T cells [86].

Different bioactive peptides have been identified in various corn breeds with antioxidant, antihypertensive, hepatoprotective, alcohol (ethanol) metabolism facilitator, anti-inflammatory, anticancer, antimicrobial and dipeptidyl peptidase IV inhibitor (antidiabetic activity) activities [97].

The suggested mechanism of action of maize peptides on liver health encompasses the inhibition of NF-κB, Fas, FasL and caspase 3, resulting in a decrease in the apoptotic process in structural and functional liver cells, thus preventing liver damage that could progress to liver injury. A role of corn bioactive peptides in the activation of Bcl-2, which inhibits apoptosis, has also been elucidated. Corn peptides could also increase the levels of endogenous enzymatic antioxidants such as superoxide dismutase (SOD), reduced glutathione (GSH) and glutathione peroxidase (GPx), which would reduce the proinflammatory environment in the liver, as well as in other organs and systems. This is due to their ability to neutralize and inhibit reactive oxygen species (ROS) with proapoptotic and pyroptotic potential [97].

Bioactive peptides from different maize species can also facilitate ethanol and other alcohol metabolisms by activating the MEOS-CYP2E1 (Microsomal Alcohol Oxidation System–cytochrome P2E1) enzyme complex, which includes the enzyme alcohol dehydrogenase, as well as enhancing and increasing the availability of NAD. All this would lead to a higher conversion of alcohols into acetate (a two-carbon fatty acid), which can be easily metabolized by the liver without generating ROS or damage to Kupffer cells [97].

The anti-inflammatory activity of corn bioactive peptides is important, which is also closely related to their antimicrobial capacity. Some peptides obtained by the cleavage of corn proteins, mainly zein (a prolamin similar to gluten gliadin), have demonstrated a TNF-α (tumor necrosis factor alpha) inhibitory capacity, which would generate an inhibition in the expression of ICAM-1 (intercellular adhesion molecule 1), MCP-1 (monocyte chemotactic protein 1) and VCAM-1 (vascular cell adhesion protein 1), which are protein molecules related to inflammation and various pathologies of the cardiovascular system, including atherosclerosis. All this would lead to a decrease in macrophage adhesion to the epithelium, as well as a decrease in proinflammatory interleukins such as IL-1 and IL-6, since an efficient inflammatory response could not be mounted. There would also be a decrease in the cyclooxygenase-2 (COX-2) pathway, so there would be a decrease in the production of proinflammatory leukotrienes, prostaglandins and thromboxanes, as well as an infra-regulation in the expression of resistin. It is worth mentioning that this signaling cascade would also decrease the nitrosative stress caused by the nitric oxide pathway. The modulation of these proinflammatory pathways has a beneficial systemic effect in different diseases, among them: metabolic endotoxemia generated by the passage of lipopolysaccharides (LPS), originating from the membrane of the resident Gram-negative bacteria of the intestinal microbiota, into the systemic circulation; type 2 diabetes mellitus; various nephropathies; liver diseases; cancer; severe acute inflammatory syndromes; dyslipidemias; cardiovascular and cerebrovascular diseases; among other conditions [88,97].

Bioactive peptides can cross the intestinal lumen by various mechanisms; the mechanism used depends mainly on the length of the peptide (the amount of amino acid residue), as well as its electrical charge. Among the transport mechanisms in the intestinal lumen are passive transport (by concentration gradient in free amino acids), proton transport coupled to PepT1 (used by dipeptides and tripeptides), sodium transport coupled to SOPT1 and 2 (used by peptides of five or more amino acids), transcytosis (used by decapeptides and longer peptides) and paracellular transport (used by free amino acids and peptides of four to nine amino acid residues) [88,97]. However, much remains to be elucidated regarding their transport, distribution and metabolism mechanisms. The study of bioactive peptides obtained from corn is in its early stages; therefore, further characterization of these bioactive peptide compounds is needed. Table 5 shows the most studied biological effects of bioactive peptides in humans from clinical trials and systematic reviews with meta-analyses.

## 5. Resistant Starches and Inflammation

Resistant starch (RS) is a linear or branched polysaccharide consisting of glucose molecules linked by glycosidic bonds, which is resistant to digestion by human amylase enzymes; it is often considered a type of dietary fiber. It is among the recent focuses of non-digestible carbohydrate therapies. Boosting intestinal butyrate production has been the focus of several RS intervention studies associated with aging [103], insulin resistance [104], metabolic syndrome, kidney disease [105] and schizophrenia [106] and may be especially relevant for diseases characterized by dysregulated epithelial integrity and immune function, such as inflammatory bowel disease [107]. The surface microstructure of a starch is the main factor affecting its digestibility. The relative amylose content, amylopectin branched chain density and crystallinity appear to influence the size, type and packing density of the blocks, which then determine the texture and porosity of the granule surface. Retrogradation and cross-linking modify starch surface crystallinity and intermolecular networks, respectively, which increases resistance to hydrolysis. It is unclear whether the blocks simply affect the surface area and integrity or whether they constitute “discrete structures” [108] that complement amylase active sites. These questions relate to whether different bacteria preferentially degrade certain starches more than others based on binding site availability or the recognition of discrete microstructures [109].

Resistant starch (RS) is a common natural component of several types of foods, including pigmented and unpigmented corn, which can be classified into four based on their physical and chemical characteristics. RS1, which is generally found in whole grains and legumes, is a starch trapped in a non-digestible matrix [110]. RS2 refers to non-gelatinized starch granules, such as starch from raw potato and high-amylose corn starch. Traditional corn starch is composed of 25% amylose and 75% amylopectin and resistant starch (1.4–39.4%) varies greatly among corn breeds [111]. High-amylose corn is rich in amylose (up to 70% of all carbohydrates) [2]. The FDA has approved Hi-Maize resistant starch (produced naturally from modified high-amylose corn) for use in patients with type 2 diabetes [112]. RS3 consists of starch that has already undergone retrogradation (starch is cooled after gelatinization). RS4, found in bread, includes starch that is chemically modified by the addition of ester or ether groups. RS is found in considerable amounts in pigmented corn, depending on the breed, variety and the processing the corn has received [109]. The correlation between SR consumption, gut health, inflammatory markers, insulin response and lipid metabolism has been well documented [113].

RS should be considered for elderly diets because it can increase the population of beneficial bacteria and butyrate production according to multiple studies. In a serial study, MSPrebiotic^®^ (Carberry, Canada), a commercial RS containing 70% RS2, promoted bifidobacteria growth and ameliorated dysbiosis related to high proteobacteria abundance in subjects ≥70 years old [103]. Consequently, changes in the levels of inflammatory markers (IL-10, C-reactive protein and TNF-α) in blood were observed. However, other research has shown that although RS was able to reduce dysbiosis in the elderly, the inflammatory levels remained elevated at the end of the study [113,114].

Beneficial effects of RS were also found in a study with 18-month-old mice that reported therapeutic effects of RS2 against high-fat-diet-induced and aging-related dysfunctions [112]. According to this study, RS2 effectively decreased the expression of systemic endotoxemia and proinflammatory cytokines, as evidenced by lower levels of serum and fecal lipopolysaccharide (LPS), which are endotoxic components in the cell membrane of Gram-negative bacteria that induce an inflammatory response mediated by colonic IL-2 and hepatic IL-4, among others. This corroborated the anti-inflammatory properties of RS2 against chronic low-grade inflammation related to aging. RS2 also improved the intestinal barrier function, which was characterized by the increased expression of colonic type 2 mucin at both mRNA and protein levels. Similarly, this study revealed that RS2 reduced the abundance of pathogenic taxa associated with obesity, inflammation and aging, such as *Desulfovibrio* (phylum *Proteobacteria*), *Ruminiclostridium*, *Lachnoclostridium*, *Helicobacteria*, *Oscillibacter*, *Alistipes*, *Peptococcus* and *Rikenella* [112,113]. Table 6 shows the most studied biological effects of retained starches in humans, based on clinical trials and systematic reviews with meta-analyses.

## 6. Lipids

Lipids are a broad group of naturally occurring molecules that include fats, waxes, sterols, fat-soluble vitamins (such as vitamins A, D, E and K), monoglycerides, diglycerides, phospholipids and others. The functions of lipids include energy storage, signaling and acting as structural components of cell membranes. Lipids have applications in the cosmetic and food industries, as well as in nanotechnology. Lipids can be broadly defined as small hydrophobic or amphiphilic molecules; the amphiphilic nature of some lipids allows them to form structures such as vesicles, multilamellar/unilamellar liposomes or membranes in an aqueous medium. Biological lipids originate in whole or in part from two distinct types of biochemical subunits or “building blocks”: ketoacyl and isoprene groups [120].

Corn germ contains more than 85% of the lipids associated with whole grains and significant amounts of triacylglycerols rich in linoleic and oleic acids, as well as phospholipids such as phosphatidyl choline, phosphatidyl inositol, phosphatidyl ethanolamine and phosphatidyl serine. Phospholipids help maintain good cell membrane integrity and proper brain function. In addition, crude corn oil contains relevant amounts of tochol derivatives, such as tocopherols and tocotrienols, which are responsible for vitamin E activity [121] and are substances considered to be the second most important defense mechanism against free radicals and oxidative stress [13].

With respect to oils from pigmented (blue and red) corns, fatty acid profiles have been reported in the following percentage ranges for blue corns: palmitic acid (C16:0), 12.50% to 16.82%; stearic acid (C18:0), 3.01% to 4. 06%; total saturated fatty acids, 15.51% to 20.88%; oleic acid (C18:1), 40.88% to 42.21%; linoleic acid (C18:2), 36.47% to 40.22%; linolenic acid (C18:3), 0.91% to 1.39%; total unsaturated fatty acids, 78.74% to 83.34%, ratio oleic: linoleic, 1.05 to 1.12 [122]. Meanwhile, the following ranges of fatty acid distribution percentages have been reported for red corn: palmitic acid (C16:0), 12.80% to 14.16%; stearic acid (C18:0), 3.14% to 3.32%; total saturated fatty acids, 15. 94% to 17.48%; oleic acid (C18:1), 33.55% to 42.84%; linoleic acid (C18:2), 39.17% to 46.48%; linolenic acid (C18:3), 0.94% to 0.96%; total unsaturated fatty acids, 80.97% to 82.97%, oleic: linoleic ratio, 0.72 to 1.09 [122]. It is noteworthy to mention that the variation among breeds and varieties is not so wide. However, climatic, geographic and soil conditions significantly affect lipid content and composition, as well as that of other macro- and micronutrients. The higher average content of oleic acid (omega-9) in blue corn, as well as its relationship with linoleic acid (omega-6), could make it a better prospect than red, white and yellow corn for the utilization of its oil, since omega-9 fatty acids have anti-inflammatory properties, while omega-6 fatty acids have proinflammatory properties by favoring the metabolic pathway of prostaglandin, thromboxane and proinflammatory leukotrienes synthesis, as well as resistin [123].

These potent antioxidants prevent the oxidation of lipids, polyunsaturated fatty acids and LDL cholesterol, the accumulation of which exacerbates cardiovascular diseases [13]. Corn contains 0.03 to 0.33% of tocopherols in its oil and its α- and γ-tocopherol forms are the most abundant [124]. The main function of these compounds is to prevent oxidative stress and the oxidation of linoleic acid (18:2 Δ9,12). Kornfeldt and Croon (1981) [125] evaluated the content of four types of corn (two normal—not rich in oil or lysine—one high in oil and one classified as opaque 2 with a high content of the amino acid lysine) and concluded that 70 to 80% of the tocopherols are associated with the germ and 11 to 27% with the endosperm. As expected, the oil-rich genotype showed the highest amounts of α- and γ-tocopherols [13].

## 7. Phytosterols: Sterols and Stanols

Phytosterols are phytosteroids, similar to cholesterol, that serve as structural components of plant biological membranes. They encompass plant sterols and stanols [126]. Corn is rich in phytosterols such as sitosterol, campesterol and stigmaesterol. According to Kornfeldt and Croon (1981) [125], corn oil contains 1441, 62 and 54 mg/100 g of the sterols 4-desmethylesterol, 4-monomethylesterol and 4.4-dimethylesterol, respectively. Among the desmethylesterols, sitosterol is the predominant form (60 to 70%), followed by campesterol (16 to 22%) and stigmaesterol (4 to 10%). Since these compounds have a similar structure to the cholesterol present in animal tissues, the consumption of phytosterols inhibits cholesterol absorption in intestinal epithelial cells [13].

Therefore, phytosterols are hypocholesterolemic compounds that help prevent cardiovascular diseases. It has been established that the daily consumption of 1 to 3 g of phytosterols reduces blood cholesterol by 5 to 20%. It is worth mentioning that the average daily consumption is between 200 and 400 mg (White and Weber, 2003) [124]. According to Raicht et al. (1980) [127] and Fiala et al. (1985) [128], the supplementation of 0.2% β-sitosterol in diets for laboratory animals caused a significant decrease in the incidence of chemically induced colon cancer tumors. What happens to phytosterols during the sequential steps of nixtamalization for tortilla production has not yet been studied. Since pericarp and germ tissues are partially lost in this process, it is to be expected that nixtamalization significantly affects the amounts and bioavailability of these important compounds [13].

## 8. Phospholipids in Corn

Corn germ is rich in amphiphilic molecules called phospholipids. Corn germ is high in lecithin, which is a high-quality additive component that exhibits advantageous interfacial properties and has attracted increasing interest as a natural emulsifier in the food, pharmaceutical and cosmetic industries [129]. It comprises a concentrated mixture of phospholipids such as phosphatidylcholine, phosphatidylethanolamine, phosphatidylinositol, phosphatidic acid and phosphatidylserine [61].

Studies in children indicate that dietary phospholipids would be better absorbed than triacylglycerols (TGs) [130]. Phosphatidylcholine, phosphatidylethanolamine, phosphatidylinositol and phosphatidylserine are the important phospholipids in maize that regulate brain function and are essential for cell membrane function [131]. Phospholipids reduce lipid levels in the liver by disrupting the absorption of sterols in the intestinal cavity. The other functions of phospholipids include the stimulation of bile acid and cholesterol secretion. Phosphatidylinositol and serine reduces blood triacylglycerols, fatty liver, bipolar disorders and neurodegenerative diseases [131]. Since corn germs are rich in phospholipids, they are a potential source for development as a functional ingredient [61].

Ninety percent of phospholipid digestion occurs at the intestinal level due to the activity of two enzymes from the pancreas: phospholipases A1 and A2. There is a wide range of effects of phospholipids on human health, for example: it has been described in several studies that natural phospholipid preparations are able to stabilize cell membranes against oxidation by replacing membrane fatty acids [132]. During various liver conditions, it has been observed that there is a reduction of phospholipids in cell membranes at the hepatic level and that when supplemented with phospholipids derived from soy or cow’s milk, phospholipids can be directly incorporated at this level, reducing alcohol-induced liver injury and lipid accumulation [133]. Several interventional authors have observed the effect of phospholipids in reducing plasma total cholesterol levels, of particular relevance in patients with primary hyperlipidemia and diabetes mellitus [134,135]. Phospholipid supplementation with krill oil significantly reduced total cholesterol, LDL cholesterol, TGs and increased HDL40 cholesterol levels, which did not occur when supplemented with fish oil, as this supplement only decreased blood TGs [136].

Studies conducted with soy phospholipid supplements showed that they are effective in reducing stress, improving memory, motility and cognition, decreasing neurodegeneration in patients with Alzheimer’s disease and improving parameters associated with concentrations in humans and animals [137,138,139]. The administration of marine phospholipids, extracted from squid (*Loligo vulgaris*) and starfish (*Asterias rubens*) meal, has been found to inhibit the progression of chemically induced colon cancer *in vitro* [140]. In addition, rats fed with phospholipids abundant in EPA and DHA showed a significant increase in the rate of apoptosis in cancer cells induced to colon carcinogenesis with 1,2-dimethylhydrazine [130,141].

## 9. Policosanols

Policosanol is the generic term for a mixture of long-chain alcohols extracted from plant waxes [142]. Policosanols are very long chain alcohols with main carbon chains ranging from 24 to 34 carbons [143].

Corn is also an important source of waxy compounds known as policosanols, which are waxes primarily associated with the epicarp and germ. Policosanols consist of a mixture of long-chain alcohols, such as hexacosanol (26:0), octacosanol (28:0), triacontanol (30:0) and docatriacontanol (32:0). According to Hwang et al. (2005) [144], corn contains approximately 10 mg/100 g of policosanols. According to Arruzazabala et al. (1994, 1996) [145,146], policosanols reduce blood lipid levels as well as platelet aggregation related to cardiovascular diseases [13].

Policosanol produces pleiotropic effects, among which its antiplatelet action, experimentally and clinically demonstrated, stands out, which is accompanied by a reduction of TXA2 plasma levels and a tendency to increase PGI 2. It has been shown that policosanol inhibits COX-1 activity, which could be the basis of its antiaggregant action. Also, experimental and clinical studies have shown that policosanol produces antioxidant effects and reduces circulating endothelial cells in plasma. It was demonstrated that single doses of this compound (10–50 mg) significantly and modestly (<20%) inhibited platelet aggregation to epinephrine and adenosine diphosphate (ADP) in healthy volunteers, while 20 mg/day for 7 days reduced aggregation by epinephrine (22.5%), ADP (21%) and collagen (11.6%) [147]. A lower dose (10 mg/day) for a longer time (14 days) inhibited aggregation by arachidonic acid (25.8%), epinephrine (17.8%) and collagen (16.0%). Arruzazabala et al. identified that policosanol (20 mg/day) administered for 7 days was more effective in inhibiting aggregation to epinephrine (32.6%) and ADP (37.3%) than acetylsalicylic acid (100 mg/day), which inhibited more aggregation to collagen (61.4%), inhibiting aggregation by epinephrine (21.9%) but not ADP. Policosanol + acetylsalicylic acid therapy markedly reduced aggregation to collagen (71.3%), epinephrine (57.5%) and ADP (31.0%) [148,149]. Due to these effects on human physiology, policosanols could be used as therapeutic agents in a large number of inflammatory-based pathologies, among them COVID-19.

In a study conducted with male Wistar rats, the consumption of a high-fat diet (HFD) caused profound hepatotoxicity evident by hepatic oxidative stress, increased serum glutamic-oxaloacetic transaminase (SGOT), serum glutamic-pyruvic transaminase (SGPT), alkaline phosphatase (ALP) and bilirubin. Treatment with policosanol (100 mg/kg) markedly reduced elevated SGOT, SGPT and ALP levels in HFD-fed rats. Similarly, policosanol significantly reduced hepatic oxidative stress manifested by reduced malondialdehyde (MDA) and increased the glutathione (GSH) level. Treatment with policosanol (100 mg/kg) was found to be more active in attenuating HFD-induced hepatotoxicity compared to policosanol (50 mg/kg) and atorvastatin (30 mg/kg). The researchers also observed that the hepatoprotective potential of policosanol was comparable to that of silymarin [142].

## 10. Conclusions

Pigmented corn has great potential for extracting and obtaining a large number of bioactive compounds with the capacity to prevent and treat a wide variety of pathologies and disorders of high prevalence and incidence. However, their cultivation and production are limited to a few geographical areas due to the high preference for higher yielding corn, such as yellow and white, many of which are genetically modified organisms, mainly the yellow varieties. The study of the consumption of pigmented corn and its bioactive compounds is an area of study with great scope for the future. However, at present, there are few and limited studies that measure the effects of pigmented corn consumption on human health, since there is a low number of clinical trials conducted with pigmented corn and/or its products and by-products, which makes it an area of opportunity for the development and evaluation of new drugs, medicines, nutraceuticals, supplements and functional foods. The benefits of pigmented corn on human health go beyond its high antioxidant capacity, as many of its compounds can act as transcription factors in the regulation of a large number of genes related to the coding of antioxidant enzymes, the regulation of signaling pathways related to aging, insulin resistance, obesity, inflammation, oxidative stress, among others. Likewise, pigmented maize has the potential to regulate the intestinal microbiota and its metabolites, as well as to chelate metal ions and toxins from the diet. For didactic and synthesis purposes, we summarize in Table 7 the potential therapeutic effects in humans of the biologically active compounds found in pigmented corn, as reported in the scientific literature.

## Figures and Tables

**Table 1:** Chemical and nutritional composition and antioxidant capacity of different corn colors.

Parameters	Raw Kernels of Blue, Purple and Black Corns	Raw Kernels of Blue, Purple and Black Corns	Tender Kernels of Sweet Yellow Corns
Humidity (%)	7.4–12.5	10.4	76
Lipids (%)	0.5–4.3	4.7	1.35
Fiber (%)	5.4–8.4	6.2	2
Ashes (%)	0.3–2.3	1	0.62
Protein (%)	7.8–8.3	9.4	3.27
Carbohydrate (%)	67.3–84.1	74.3	18.7
Starch (%)	64–71	59–65	5.7
Caloric value (kcal/100 g)	353.9–364	365	86
Tannins (mg/g)	0.09–0.24	ND	NA
Phytic acid (mg/g)	0.55–1.6	ND	NA
Antioxidant activity (%)	9.73–18.75	ND	NA
Total phenolic content (mg GAE/100 g)	35.7–61	34.7–47.2	45.5–51.1
Total anthocyanins (mg CGE/kg)	516.3–543.9	2.8–13.3	2.9–5.7
Beta-carotene (µg/100 g)	ND	ND	47
Alpha-carotene (µg/100 g)	ND	ND	16
Beta-cryptoxanthin (µg/100 g)	ND	ND	115
Vitamin A (IU)	ND	ND	187
Lutein + zeaxanthin (µg/100 g)	ND	ND	644
Copper (ppm)	1.4–2.33	3	0.54
Manganese (ppm)	0.64–5.4	5	1.63
Iron (ppm)	3.6–17.4	27	5.2
Zinc (ppm)	0.64–22.4	22	4.6
Calcium (ppm)	43–72	70	20
Magnesium (ppm)	941–1100	1270	370
Phosphorus (ppm)	2630	2100	890
Potassium (ppm)	3810	2870	2700
Sodium (ppm)	50	350	150
Selenium (ppm)	0.022	0.155	0.006
Thiamine (ppm)	1.6	4	1.55
Riboflavin (ppm)	2.3	2	0.55
Niacin (ppm)	26	36	17.7
Pantothenic acid (ppm)	5.5	4	7.17
Pyridoxine (ppm)	4.7	6	0.93

CGE: cyanidin-3-O-glucoside equivalents; GAE: gallic acid equivalents; ND: not determined; ppm: parts per million; IU: international units. The content of bioactive compounds such as anthocyanins can vary greatly among different breeds and varieties of corn, even among those of the same colors. The table shows the constituents and contents usually reported for corn, as well as their highest frequency ranges.

**Table 3:** Biological effects of phenolic compounds and anthocyanins found in pigmented corn in humans from clinical trials and systematic reviews with meta-analyses.

Bioactive Compound	Description of the Study	Results	References
Purified anthocyanins	Randomized placebo-controlled clinical trial: 169 participants with dyslipidemia were randomly assigned to placebo (*n* = 43), anthocyanins 40 mg/day (*n* = 44), 80 mg/day (*n* = 40) or 320 mg/day (*n* = 42) for 6 and 12 weeks.	↑ T-SOD. ↓ IL-6, TNF-α, 8-iso-PGF_2α_, MDA, CRP. ↑ Antioxidant capacity and anti-inflammatory responsiveness in subjects with dyslipidemia.	[41]
Purified anthocyanins	Randomized placebo-controlled clinical trial: 206 persons aged 60 to 80 years, diagnosed with mild cognitive impairment (MCI) or two or more cardiometabolic disorders (diabetes, hypertension, obesity) in Norway. Given 320 mg/day or placebo for 4 weeks.	Improved episodic memory on an online cognitive test battery (CogTrack) after 8 weeks of anthocyanin administration (*p* = 0.007).	[64]
Anthocyanins in blueberries	Randomized controlled clinical trial: daily supplementation with blueberries in subjects aged 50–65 (men and women) years with subjective cognitive impairment.	↑ Lexical ability/access (*p* = 0.003), memory (*p* = 0.04), correction of peripheral hyperinsulinemia (*p* = 0.04). Improvement in executive capacity.	[65]
Anthocyanins from various dietary and supplemental sources	Systematic review with meta-analyses of RCTs: in subjects with cardiometabolic disorders and type 2 diabetes mellitus, 32 RCT (1491 participants).	↓ Fasting glucose, 2 h postprandial glucose, glycosylated hemoglobin, TC and LDL-C. Significant improvements in metabolic control of lipids and glycemia.	[49]
Polyphenols from cherry juice	Systematic review with meta-analyses of RCTs: 10 RCTs were included.	↓ Fasting glucose. No significant effects on TG, TC, HDL-C, LDL-C, BMI and insulin.	[52]
Polyphenols from dietary intake	Systematic review with meta-analyses: Polyphenols in the treatment of NAFLD. From RCTs of curcumin, resveratrol, naringenin, anthocyanins, hesperidin, catechin, silymarin and genistein (2173 participants).	↓ BMI, AST, ALT, TG, TC, HOMA-IR, NAFLD grade, LDL-C. ↑ HDL-C. Overall improvement of NAFLD.	[51]
Anthocyanins found naturally in food matrices	Systematic review with meta-analyses: influence of dietary anthocyanins on Firmicutes/Bacteroid (Fir/Bac) and SCFA content.	↑ Fecal acetic acid, propionic acid and butanoic acid. Improvement in Fir/Bac ratio. ↓ Obesity-induced intestinal dysbiosis.	[66]
Supplementation with ferulic acid	Double-blind, placebo-controlled RCT: Measurement of changes in lipid profile, oxidative stress and inflammatory status in subjects with hyperlipidemia. Given 1000 mg ferulic acid for six weeks in treatment group (*n* = 24) and placebo group (*n* = 24).	↓ TC (*p* = 0.001), LDL-C (*p* < 0.001), oxidized LDL-C (*p* = 0.002), TG (*p* = 0.049), MDA (*p* < 0.001), hs-CRP (*p* < 0.001) and TNF-α (*p* < 0.001). ↑ HDL-C (*p* = 0.045).	[67]
Dietary supplement based on beer bagasse extract with 91.3 mg/100 g of bioaccessible ferulic acid	Crossover clinical trial: 40 normoglycemic subjects divided into two groups (treatment group and control group).	Significantly lower postprandial blood glucose values after 90 and 120 min of glucose consumption. Similar values after 60 min of glucose administration.	[68]
Ferulic acid from a variety of dietary and supplemental sources	Systematic review with meta-analyses of preclinical studies: animal models with Alzheimer’s disease, 344 animals in 12 preclinical studies.	Improved spatial memory capacity of rodents in MWM and Y maze experiments (*p* < 0.005). ↓ Deposition of β-amyloid in the brains of animal models (*p* < 0.005). Antiamyloidogenic, anti-inflammatory, antioxidant, mitochondrial protection and apoptosis inhibition effects.	[69]

ALT: alanine aminotransferase. AST: aspartate aminotransferase. BMI: body mass index. CRP: C-reactive protein. HDL-C: high-density protein-bound cholesterol. HOMA-IR: homeostatic model for assessing insulin resistance. hs-CRP: high-sensitivity C-reactive protein. IL-6: interleukin 6. iso-PGF_2α_: iso-prostaglandin F_2α_. LDL-C: low-density lipoprotein cholesterol. MDA: malondialdehyde. NAFLD: nonalcoholic fatty liver disease. RCT: randomized clinical trial. SCFA: short-chain fatty acids. TC: total cholesterol. TG: triacylglycerols. TNF-α: tumor necrosis factor α. T-SOD: total superoxide dismutase. ↓: decrease. ↑: increase.

**Table 4:** Biological effects of carotenoids (carotenes and xanthophylls) in humans from clinical trials and systematic reviews with meta-analyses.

Bioactive Compound	Description of the Study	Results	References
Food supplement based on 14 mg of zeaxanthin and 7 mg of lutein.	Double-blind, placebo-controlled RCT. Six-month duration and 36 participants (26 men and 7 women). *n* = 24 supplemented group and 9 placebo group.	Macular pigment optical density increased in the right eye (*p* < 0.001) and in the left eye (*p* > 0.05). Significant improvements in contrast sensitivity with glare in both eyes (*p* = 0.02 and 0.01). Improvements in monocularly tested glare recovery time (*p* = 0.008 and *p* = 0.02). Decrease in preferred luminance required to complete visual tasks (*p* = 0.02 and 0.03). Improvements in divided attention UFOV scores (*p* < 0.001) and accident risk composite score (*p* = 0.004).	[80]
Different kinds of carotenoids in different formulations, presentations and doses in the delay and prevention of dementia and mild cognitive impairment.	Systematic review with meta-analyses. Articles included until 23 February 2023. Included 23 studies (*n* = 6610) involving 1422 patients with dementia, 435 patients with mild cognitive impairment and 4753 controls.	Dementia patients were shown to have lower blood levels of lycopene, α-carotene, β-carotene, lutein, zeaxanthin and β-cryptoxanthin than controls. A similar stable relationship between blood carotenoid levels and mild cognitive impairment was not observed.	[81]
Food supplement with 22 mg carotenoids (10 mg lutein, 10 mg mesozeaxanthin and 2 mg zeaxanthin), 1 g fish oil and 15 mg vitamin E.	RCT of 24-month duration. *n* = 60 (30 treatment group vs. 30 placebo group). Cognitively healthy men and women aged ≥ 65 years were included.	Fewer errors in working memory tasks. ↑ tissue carotenoid concentrations, serum xanthophyll carotenoid concentrations and plasma ω-3 fatty acid concentrations. As the cognitive load of working memory tasks increased, the treated group outperformed the placebo group. Increased nutritional intake of carotenoids and ω-3 fatty acids may be beneficial in reducing cognitive decline and the risk of dementia in old age.	[82]
Oral supplementation with β-carotene from various sources and different doses.	Systematic review with meta-analyses of RCTs on cardiovascular disease. Articles included from the year 1900 to March 2022. Ten trials and 16 reports were included in the meta-analyses (*n* = 182,788 persons).	Supplementation with β-carotene slightly increased overall cardiovascular incidence (RR: 1.04) and was consistently associated with increased cardiovascular mortality (RR: 1.12). When β-carotene treatments were administered alone, an increased risk of cardiovascular outcomes was observed (RR: 1.06). No apparent effects on cardiovascular health.	[83]
Oral supplementation with lutein, zeaxanthin and long-chain polyunsaturated fatty acids (ARA, EPA and DHA).	Double-blind, placebo-controlled RCT with parallel groups in healthy senile Japanese individuals with memory impairment. Duration of 24 weeks. Memory functions were assessed using Cognitrax before and after the intervention.	The formulation did not significantly affect memory function in healthy, non-demented, memory-impaired elderly persons, whereas it improved memory function in healthy, non-demented, cognitively impaired elderly persons.	[84]

ARA: arachidonic acid. DHA: docosahexaenoic acid. EPA: eicosapentaenoic acid. g: gram. mg: milligram. RCT: randomized clinical trial. RR: relative risk. UFOV: Useful Field Of Vision. ↑: increase.

**Table 5:** Biological effects of bioactive peptides in humans from clinical trials and systematic reviews with meta-analyses.

Bioactive Compound	Description of the Study	Results	References
Dietary supplement of a fish protein hydrolysate (Gabolysat^®^, (Aras, France)), magnesium and vitamin B6.	Observational, multicenter, prospective, longitudinal, prospective study in patients diagnosed with anxiety (≥20 on the Hamilton Anxiety Rating Scale). A 28-day intervention.	A 50% reduction in Ham-A score for 41.9% of patients. Mean Ham-A score decreased 12.1 ± 5.7 points (*p* < 0.001) between time 0 (25.6 ± 3.8) and time 1 (13.6 ± 6.0).	[98]
Oral supplement of bioactive collagen peptides.	RCT over 12 weeks with 180 active men and women aged 18–30 years with exercise-related knee pain without diagnosed joint disease; 5 g of specific collagen peptides vs. placebo.	Reduced exercise-induced knee pain compared to placebo group (*p* = 0.024).	[99]
Oral supplement with 15 g of bioactive collagen peptides.	RCT for 12 weeks with 97 male athletes divided into three groups (collagen peptides vs. whey protein vs. placebo).	↑ fat-free mass (*p* = 0.01). ↓ fat mass (*p* = 0.023). Similar results between collagen bioactive peptides and whey protein.	[100]
Oral supplementation of bioactive collagen peptides, vitamin D and calcium.	RCT for three months with 51 postmenopausal women with osteopenia. Two groups (5 g collagen peptides + 500 mg calcium + 400 IU vitamin D3 vs. 500 mg calcium + 400 IU vitamin D3).	↓ P1NP by 13.1% (*p* < 0.001) and ↓ CTX by 11.4% (*p* = 0.058) within 3 months after supplementation. No adverse effects were present. The addition of bioactive collagen peptides in a calcium and vitamin D supplement may enhance their already known positive effect on bone metabolism.	[101]
Oral supplementation of bioactive peptides derived from whey protein hydrolysate.	RCT in subjects diagnosed with prediabetes. Two groups (placebo vs. treated). Alpha-glucosidase inhibitory properties.	↓ Incremental areas under the glucose curve (*p* = 0.0472) and ↓ glycosylated hemoglobin HbA1c (*p* = 0.0244). Potential to modulate postprandial hyperglycemia and, therefore, may contribute to reducing the future risk of developing DM2.	[102]

CTX: carboxy-terminal telopeptide of type I collagen. DM2: type 2 diabetes mellitus. g: gram. IU: international unit. mg: milligram. P1NP: type I procollagen N-terminal propeptide. RCT: randomized clinical trial. ↓: dismi-nution. ↑: increase.

**Table 6:** Biological effects of resistant starches in humans from clinical trials and systematic reviews with meta-analyses.

Bioactive Compound	Description of the Study	Results	References
Oral supplement of resistant starch and polydextrose.	Double-blind, placebo-controlled RCT. Oral intervention in 75 healthy subjects for 50 days.	RS increased the total number of mitotic cells within the crypt by 60% (*p* = 0.001) compared to placebo, as did SCFAs. The effects were more evident in subjects >50 years.	[115]
Resistant starch type 2.	Controlled RCT. Intervention with RS (9.6 vs. 4.7 g) in 128 healthy men and women aged 40–65 years with BMI between 18.5–39.9 kg/m2.	The RS-treated group decreased their postprandial glucose (*p* = 0.003) and postprandial serum insulin levels (*p* < 0.001).	[116]
Resistant starch from various sources.	Systematic review with meta-analyses of RCTs of 8 studies (years 2020 and 2021) and 301 individuals with chronic kidney disease on dialysis.	Favorable changes in gut microbiota richness and diversity (*p* < 0.001) and no statistical significance in fecal acetate, propionate and butyrate.	[117]
Resistant starch from various sources.	Systematic review with meta-analyses of RCTs of 13 studies (*n* = 15–75 per study) (years 1988–2019) with 4–14 weeks interventions with SR of different types and pharmaceutical presentations on various inflammatory biomarkers.	↓ serum levels of indolephenol sulfate (*p* = 0.0002), blood phosphorus (*p* = 0.03), IL-6 (*p* = 0.02) and uric acid (*p* = 0.004) in dialysis patients. No significant changes in hs-CRP, serum creatinine, BUN, blood paracresol sulfate and blood lipids.	[118]
Resistant starch type 2 from various sources.	Systematic review with meta-analyses of RCTs of 8 studies (as of October 2019).	↓ IL-6 (*p* < 0.001) and TNF-α (*p* = 0.001). No significant changes in CRP (*p* = 0.61). Changes in IL-6 were dependent on study quality and duration of intervention.	[119]

BMI: body mass index. BUN: blood urea nitrogen. CRP: C-reactive protein. DM2: type 2 diabetes mellitus. g: gram. hs-CRP: high-sensitivity C-reactive protein. IL-6: interleukin 6. RCT: randomized clinical trial. RS: resistant starch. RS2: resistant starch type 2. SCFAs: short-chain fatty acids. TNF-α: tumor necrosis factor alpha. ↓: decrease.

**Table 7:** Possible therapeutic effects in humans of the biologically active compounds found in pigmented corn, as reported in the literature.

Bioactive Compound	Effects on Human Health	References
Anthocyanins	↓ Cardiac cell damage. ↓ Incidence of neurodegenerative diseases. ↓ Acute kidney damage. ↓ Proliferation of small cell cancer. ↓ Angiogenesis in cancer. ↓ Metastatic migration. ↓ Proinflammatory state. ↓ Risk of ischemia. ↓ Excitotoxicity. ↓ Aggregation of encephalic prion proteins. ↓ ROS. ↓ Plasma lipids. ↓ Insulin resistance. ↓ Fasting plasma glucose. ↓ Glycosylated hemoglobin. ↓ Mitochondrial membrane damage. ↓ Lipid peroxidation. ↓ RNS. ↓ Risk of developing obesity. ↓ Liver damage and hepatotoxicity. ↑ Antioxidant enzymes.	[40,42,43,44]
Carotenoids	↓ Age-related macular degeneration. ↓ Risk of developing cataracts. ↓ Retinitis pigmentosa. ↓ C-reactive protein. ↓ Serum homocysteine. ↓ LDL-C. ↓ Risk of the development of type 2 diabetes mellitus. ↓ Steatohepatitis. ↓ Atherosclerosis. ↓ Dyslipidemias. ↓ UV light-induced damage. ↓ Blue light absorption ↓ Risk of developing prostate, breast, urothelial and melanoma cancers. Apoptosis modulators. ↑ Bioavailability of cis-lycopene. ↑ HDL-C. ↑ Endogenous enzymatic antioxidants. ↑ Hepatic phase II metabolism.	[150,151,152,153,154,155]
Bioactive peptides	Antioxidant capacity. ACE inhibitors. Antihypertensive activity. Antimicrobial and bacteriostatic capacity. Antidiabetic capacity. Anti-obesity capacity. Appetite modulating capacity. Mood modulating capacity. Chelating effects of heavy metals, metalloids and transition metals, as well as other environmental toxins. Anticoagulant capacity. Antithrombotic effects. Bone calcification. Anticariogenic effects. Immunomodulatory properties. Opioid activity. Anticancerogenic properties. ↓ LDL-C, total cholesterol and triacylglycerols.	[86,156,157,158,159]
Resistant starches	Prebiotic activity by fermentation by BAL. ↑ Species of the genera *Lactobacillus* and *Bifidobacterium*. ↑ Synthesis of SCFA. Appetite regulation. ↑ Peripheral glucose uptake. ↑ Intestinal motility. ↓ Glucose absorption rate at intestinal level. Immunomodulatory effects at the gastroenteric level. ↓ Risk of developing colorectal cancer. ↓ Risk of developing chronic kidney disease. Modulates the intestinal microbiota. ↓ Diarrheal symptomatology. ↓ Constipation.	[160,161,162,163,164]
Lipids, mainly linoleic acid (omega-6)	↑ Proinflammatory factors. ↑ Risk of developing cardiovascular disease. ↑ Risk of developing atherosclerotic disease. ↑ E2-series prostaglandins, proinflammatory thromboxanes, B4-series leukotrienes, 4-series lipoxins. They favor the arachidonic acid pathway. Atypical neurodevelopment in infants. ↑ Neuroinflammation. ↑ Immune system activity. ↑ Risk of dyslipidemias. ↑ Lipid peroxidation. ↑ Risk of developing some types of cancer.	[165,166,167,168,169]
Phytosterols	↓ LDL-C. Immunomodulatory activity. Antihypertensive activity. Regulates cholesterol, triacylglycerol and fatty acid homeostasis. Antidepressant activity. Anti-aging activity. Anti-oncogenic activity. ↑ Bone regeneration. Anti-osteoporotic activity. ↑ Lung protection. Modulation of the inflammatory state. Anti-adipogenic activity. Regulation of redox state. Antioxidant capacity. ↓ Decreases lipid peroxidation. Neuroprotective activity. Anti-amyloidogenic activity. Partially inhibits cholinesterases. Favorable effects on the health of athletes, as well as on sports performance.	[170,171,172,173,174]
Phospholipids	Regulation of the inflammatory state. Renewal of cellular and mitochondrial membrane lipids. ↓ Risk of developing cardiovascular diseases. Slowing of age-associated cognitive decline. Improvement in neuronal synapses. ↑ Concentration capacity. Anti-hyperlipidemic activity. ↑ Neuronal myelin sheath regeneration capacity.	[175,176,177,178,179]
Policosanols	↑ GSH. ↑ Antioxidant activity. ↓ Myocardial necrosis. ↓ Cardiac mast cells. ↓ Polymorphonuclears at cardiac level. ↓ Enteric proinflammatory factors. ↓ Hepatic lipid accumulation. ↓ Lipid peroxidation. ↓ Total cholesterol, LDL-C and serum lipids. ↓ Risk of developing metabolic syndrome. Improves venous blood return. Improves glycemic and insulin profiles. Modulates blood pressure.	[180,181,182,183,184]
Ferulic acid	Neutralization and decrease of ROS. Antioxidant effect. Improvement in endothelial function. ↓ Proliferation of smooth muscle cells at endothelial level. Antihypertensive effects. ↑ Nitric oxide availability at endothelial level. ↑ Cellular reuptake of glucose. ↑ Protein synthesis. ↑ Lipolysis. ↓ Lipogenesis. ↓ Lipid peroxidation. ↓ Protein peroxidation. Possible preventive and therapeutic use in neurodegenerative diseases (Alzheimer’s disease and Parkinson’s disease). Protective effect against UVA and UVB radiation.	[15,69,185,186,187]
Phlobaphenes	↓ Amount of mycotoxins in corn kernels. ↑ Thickness of the pericarp of pigmented corn, mainly red varieties. Its effects on human physiology are still unknown; however, it is hypothesized that they could be similar to those shown in various phenolic compounds, such as tannins.	[25,188]

SCFA: short-chain fatty acids. LAB: lactic acid bacteria. ACE: angiotensin-converting enzyme. GSH: reduced glutathione. HDL-C: high-density protein-bound cholesterol. LDL-C: low-density lipoprotein cholesterol. RNS: reactive nitrogen species. ROS: reactive oxygen species. UV: ultraviolet. UVA: ultraviolet A spectrum. UVB: ultraviolet B spectrum. ↓: decrease. ↑: increase.
